# 2466. The Evolution of Candidemia during the COVID-19 Pandemic in Connecticut

**DOI:** 10.1093/ofid/ofad500.2084

**Published:** 2023-11-27

**Authors:** Johanna Gleason-Vergados, Maria Correa, James Meek, David Banach

**Affiliations:** University of Connecticut School of Medicine, New Haven, Connecticut; Connecticut Emerging Infections Program, Yale School of Public Health, New Haven, Connecticut; Connecticut Emerging Infections Program, Yale School of Public Health, New Haven, Connecticut; UConn Health, Farmington, Connecticut

## Abstract

**Background:**

Candidemia is a life-threatening bloodstream infection. The changing epidemiology of candidemia during the COVID-19 pandemic has not been well described. The goal of this study is to describe trends in candidemia epidemiology throughout various phases of the COVID-19 pandemic in Connecticut.

**Methods:**

Using statewide surveillance we identified all adult Connecticut residents with candidemia between January 2020 and October 2022. Cases were aggregated by four-month intervals. Three surges of COVID-19 (Initial, Delta, and Omicron variant surges) were identified based on statewide COVID-19 case numbers. Incidence rate ratios (IRR) were calculated to compare the incidence rate (IR) of candidemia during surge periods to the IR of candidemia in non-surge periods (four-month intervals before and after surges). Patient demographics and clinical characteristics were compared using chi-square analysis.

**Results:**

721 candidemia cases were included in the analysis (255 in 2020, 290 in 2021, and 176 in 2022). The IR of candidemia during the Initial surge was 1.49 times higher than the 4 months after the Initial surge (95% CI: 1.09-2.03). The IR of candidemia during the Delta surge was 1.46 times higher than the preceding 4 months (95% CI: 1.07-1.99) and 1.13 times higher than the 4 months after the Delta surge (95% CI: 0.84-1.51). The IR candidemia during the Omicron surge was 1.05 times higher than the preceding 4 months (95% CI: 0.78-1.41) but 1.56 times higher than 4 months after the surge (95% CI: 1.12-2.17). During surge periods candidemia was more frequently associated with persons of non-white race/ethnicity, hospital-onset infection, mechanical ventilation and mortality (all p < 0.05).

**Figure:**
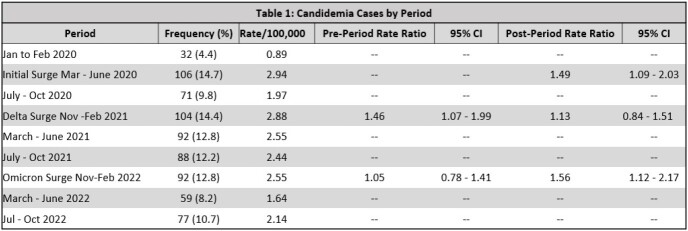

**Figure:**
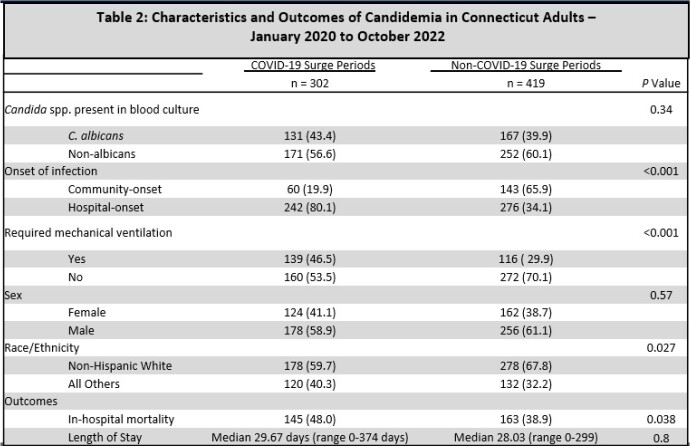

**Conclusion:**

We identified an association between COVID-19 surges and increased rates of candidemia. Candidemia cases during surge periods occurred more frequently among non-white persons, were more likely to have hospital-onset and were more severe suggesting that COVID-19 treatments or interventions may have impacted candidemia rates. Further study is needed to understand factors that resulted in different IRs of candidemia during various phases of the COVID-19 pandemic to inform strategies to prevent candidemia during future public health crises.

**Disclosures:**

**All Authors**: No reported disclosures

